# A study on the performance and cost-effectiveness of robots in replacing manual nucleic acid collection method: Experience from the COVID-19 pandemic

**DOI:** 10.1371/journal.pone.0276782

**Published:** 2022-11-03

**Authors:** Zhuoyuan Chi, Yusi Tu, Fangfang Gong, Wenxi Tang

**Affiliations:** 1 Department of Pharmacoeconomics, School of International Pharmaceutical Business, China Pharmaceutical University, Nanjing, China; 2 Center for Pharmacoeconomics and Outcomes Research, China Pharmaceutical University, Nanjing, China; 3 Shenzhen Luohu Hospital Group, Shenzhen, China; Kathmandu Institute of Applied Sciences, NEPAL

## Abstract

**Background:**

The COVID-19 pandemic has led nucleic acid collection and detection became a measure to ensure normal life in China. Considering the huge detection demand, it has emerged that robots replace manual sample collection. However, the cost-effectiveness of nucleic acid collection by robots instead of humans remain unknown.

**Methods:**

This study was approved by the Ethics Committee of the Shenzhen Luohu District People’s Hospital, number 2021-LHQRMYY-KYLL-031a. All participants signed the written informed consent of this study. 273 volunteers were recruited on December 1^st^ 2021 from Shenzhen and divided into six groups: one group to be sampled by robots and the others to be sampled manually with varying specifications for swab rotation and insertion time. Questionnaires were distributed to the robot group to ask them sampling feeling. The effectiveness and safety of sampling were evaluated through the sampling efficiency, adverse events and sampling feeling of different groups. The economics of the different methods were judged by comparing the sampling cost for each.

**Results:**

The sampling efficiency of the robot group was 96.9%, and there was no statistically significant difference between the other five manually sampled groups (p = 0.586). There were no serious adverse events in any of the six groups, but nasal soreness and tearing did occur in all group. Of the volunteers who underwent robotic sampling, 85.94% reported that the experience was either no different or more comfortable than the manual sampling. In economic terms, a single robot used to replace medical staff for sample collection becomes economically advantageous when the working time is ≥ 455 days. If multiple robots are used to replace twice the number of manual collections, it becomes more economical at 137 days and remains so as long as the robot is used.

**Conclusions:**

It appears safe and effective for robots to replace manual sampling method. Implementation of robotic sampling is economical and feasible, and can significantly save costs when working over a long term.

## Introduction

Since the outbreak of the COVID-19 pandemic, real-time quantitative polymerase chain reaction (RT-PCR) detection has been the primary detection method for diagnosing the disease because of its early diagnosis, good sensitivity, and high specificity [1–4]. The principal collection methods for nucleic acid detection are oropharyngeal and nasopharyngeal swabs [5]. Compared with oropharyngeal swabs, nasopharyngeal swabs better avoid the sample uncertainty caused by actions such as swallowing, eating, and drinking [6]. The number of institutions that can carry out nucleic acid testing in China has reached 11,581 [7], and the number of people tested exceeds 1.5 million tests per day. At present, the situation of epidemic prevention and control in China remains severe, and the epidemic situation is generally distributed in multiple locations [8]. There is still high demand for nucleic acid testing at border ports, cold chain transportation facilities, hospitals and other places [9, 10].

The current process of collecting nucleic acid test samples requires medical staff to have close contact with the tested person. If a person being tested coughs or breathes hard, it produces a large number of droplets or aerosols [11], greatly increasing the potential for cross-infection of medical staff during the sampling process. In addition, in the face of the huge demand for nucleic acid testing, medical staff must wear protective clothing for a long time to sample patients, and this working environment coupled with the work intensity brings significant psychological pressure to medical staff [12]. The use of robots to replace medical staff for nucleic acid autonomous sampling could reduce the work intensity and mental pressure [13], reduce the risk of cross-infection, concentrate limited medical resources in the treatment of acute and critical illness and reduce mortality.

**Fig 1:**
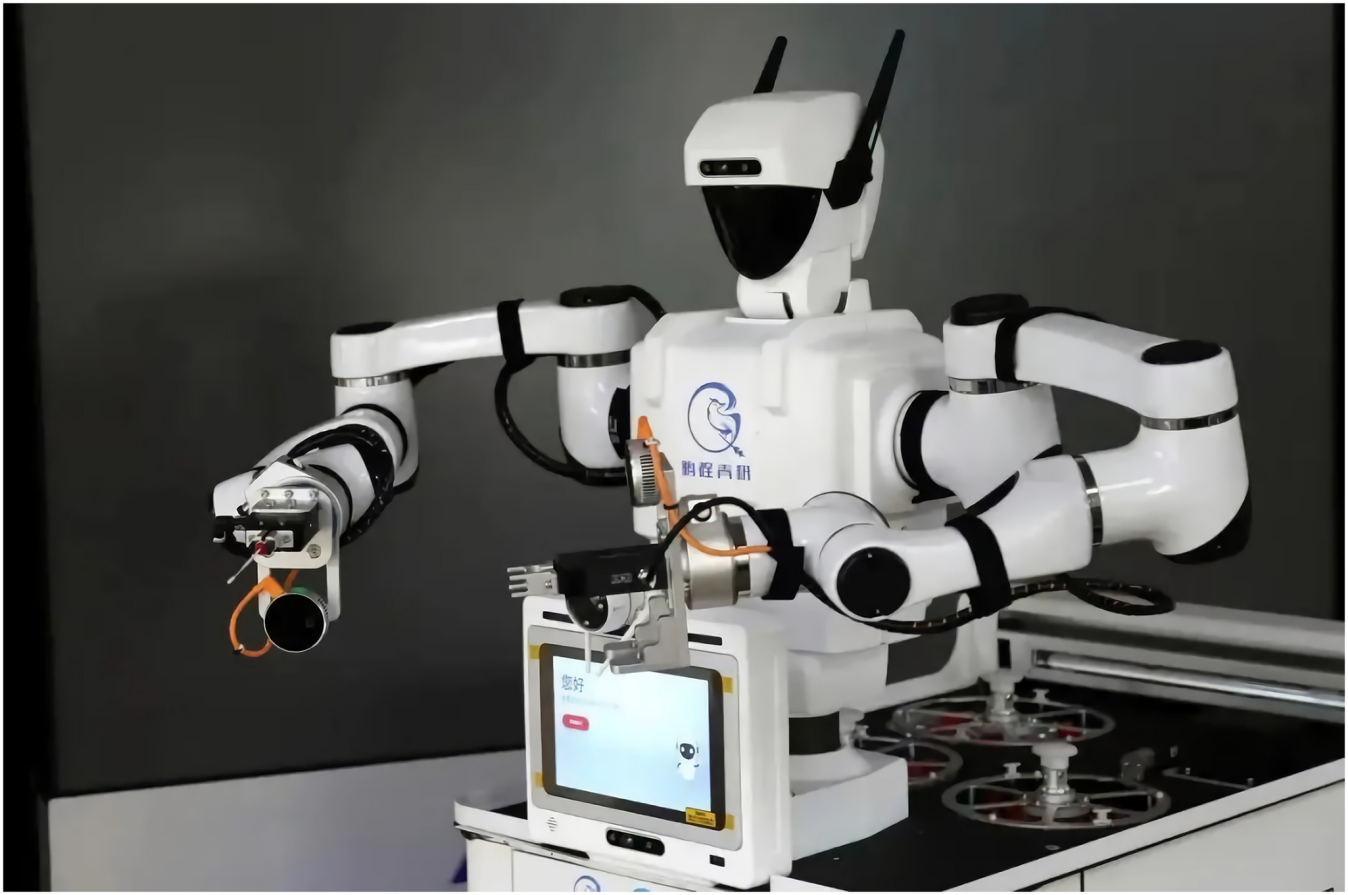
Picture of qinggeng robot.

It is not uncommon to use robots to assist in medical testing. Hamilton JR of the University of California, Berkeley, used robots to collect saliva from people and developed a set of robot programs to quickly extract samples [14]. Hangzhou Huxi Yunbaisheng Technology Co., Ltd. has developed the world’s first fully automatic nasopharyngeal swab sampling robot, named “Qinggeng”. The robot is composed of a chassis, robotic arms, sensors and other parts, with artificial intelligence technology as the core (Fig 1).

The robotic arm automatically obtains nasopharyngeal swabs under the control of the motion planning system. To ensure the accuracy of sampling, the artificial intelligence (AI) visual analysis technology used by the robot can automatically judge and identify more than 500 feature points of the face, and can capture slight movement trajectories of the person from whom the sample is being collected. At close range, it uses finite element simulation technology and high-performance probabilistic models to remove noise and background, locate the nostrils, and adjust the angle of nasopharyngeal swab collection. Because it uses light detection and ranging (LIDAR), which is insensitive to skin color and texture, it can recognize people of different physiques, ages, and skin tones. To ensure the safety of the collection process, the robot uses a high-sensitivity force-controlled robotic arm and a force-controlled sequentially encoded rotational variable operation (SERVO) system during the collection process, so that the minimum force control can reach 0.2N, comparable to that of manual collection [15]. In addition, the robot has an emergency pause button that can be used in emergencies during the collection process to protect the safety of the subject. Disposable sampling swabs and sample storage tubes are used in the for each person being sampled. The robot arm holds the nasopharyngeal swab to collect samples without touching the person to be collected. To avoid cross-infection, the sampling arm is completely sterilized after sample collection [16].

Based on the above background, we sought to explore the differences between manual swab collection and robotic swab collection in the COVID-19 era, evaluate the safety, effectiveness and cost-effectiveness of robotic sample collection.

## Materials and methods

### Ethics statement

This study was approved by the Ethics Committee of the Shenzhen Luohu District People’s Hospital, number 2021-LHQRMYY-KYLL-031a. All participants were presented with a detailed background and flow of the project, including robot constitution, sampling considerations, etc. and all participants signed the written informed consent of this study.

### Inclusion and exclusion criteria

Respondents without serious illness aged 18 or older but less than 60 years old who were able to express and understand the purpose of this research independently and voluntary agreed to participate in this research. Also, all enrolled subjects were required to have had the experience of throat swab sampling before.

Exclusion criteria were 1) failure to understand research or to articulate thoughts clearly 2) a recent history of nasal trauma or surgery; 3) obvious deviated nasal septum or chronic nasal passage obstruction; 4) a history of severe coagulation disorder; and 5) serious diseases such as malignant tumor, severe cardiovascular and cerebrovascular disease, severe Parkinson’s disease, and severe mental illness.

### Collection process

Each volunteer meeting the conditions for inclusion participated in an interview including the informed consent form explaining the purpose and process of the study, and subjects could withdraw from the study at any step without bearing the relevant costs. Volunteers were divided into six groups according to the type of sampling and the duration time for manual sampling. Volunteers in the robotic collection group were asked to complete an additional questionnaire about robotic sampling to record their perceptions of the process in Table 1.

**Table 1:** Questionnaire in robotic group.

Question description	Answers
Do you know how to collect nasopharyngeal swabs?	
Are you afraid of robotic nasopharyngeal swab collection?	
If you are afraid, what specifically are you afraid of?	
Please write down your sampling experience.	
How did it compare with manual sampling?	
Are you satisfied with this robotic swab collection operation?	

#### Robot collection

First, the sampling ID of the volunteer was registered and matched with the sampling tube. Volunteers were seated facing the sampling robot, the robot used AI visual analysis to identify and correct for the position of the volunteer’s face. After successful recognition, the robotic arm inserted a cotton swab into the nasopharynx of the sampled person for sampling [3]. The sampling process was set for the collection swab to rotate three times in the nasal cavity and remain in place for 5 seconds. After sampling, the robotic arm removed the swab from the nasal cavity and placed it into the sampling tube, rotated and tightened the sampling tube, and prompted the volunteer to complete the sampling. Then, the sampling robot automatically sterilized the equipment via infrared induction and to prepare for the next volunteer.

#### Manual collection

Senior nurses were trained on the anatomy of the nasopharynx by one or two ear, nose and throat (ENT) experts and senior ENT nurses. Samples were collected from the enrolled volunteers according to the nasopharyngeal swab sampling process video published by the New England Journal of Medicine [17]. During manual sampling process, the sampling ID of the volunteer was first registered and matched with the sampling tube. The volunteers naturally relaxed and fully raised their heads facing the medical staff. The medical staff first fully infiltrated the cotton swab in the sampling solution, and then put the swab against the nostril wall and slowly turned it into the volunteer’s nasopharynx, and then removed it slowly, wiping while rotating [18]. The manual sampling process was specified be collected in one of five patterns: rotate three times over 5 seconds, rotate three times over 10 seconds, rotate three times over 15 seconds, rotate five times over 10 seconds, or rotate five times over 15 seconds. After the sampling, the medical staff disinfected their hands with alcohol and prepared to sample the next volunteer.

### Data information

Basic information such as gender, age, education etc. was collected to compare baseline characteristics between different groups. Research related information mainly included samples CT value, which is used to judge whether the collected nucleic acid sample is qualified. Sample Ct values of less than the internal standard Ct value is judged to be valid; higher values are considered invalid [19]. The kits used for the collection and testing swabs were from the new coronavirus 2019-nCoV nucleic acid detection kit (fluorescent PCR method) produced by Shanghai Berger Medical Technology Co., Ltd, significantly, the internal standard CT value in this product is 30. Cost information include upfront cost and sampling cost.

#### Upfront cost

The initial cost of manual sampling includes a series of related expenses such as pre-job training and certification of medical staff [20]. These expenses can be ignored if they are evenly distributed to individuals. The upfront cost of robot sampling is research and development (R&D) expenditure, that is, a series of expenditures such as personnel fees, labor fees, and material losses incurred in the research process [21]. According to the company’s introduction, the R&D expenditure of a Qinggeng robot is 500,000 CNY (78300USD, 1CNY = 0.16USD), including the R&D expenditure of the visual force control algorithm and the overall project integration R&D expenditure.

#### Sampling cost

The sampling cost of manual sampling includes the following three parts: 1) The construction expenditure of the sampling booth; 2) the hygiene materials required by medical staff, including sampling swabs, protective clothing, and disinfection supplies [22]; and 3) subsidies for medical staff. The sampling cost of robotic sampling is divided into the following 3 parts [23]: 1) fixed costs, including the construction and installation expenses of production bases, assembly lines and other production facilities; 2) variable costs such as manufacturing costs of robot components and costs of sanitary materials, such as swabs, protective films, and disinfectants; and 3) depreciation costs [24].

According to the company’s quotation, the material cost of a Qinggeng second-generation robot is about 630,000 CNY (99162USD), including part composition and depreciation costs. The service life of a machine is 20,000 to 30,000 hours. Based on performing nucleic acid sampling work 24 hours a day, the estimated usable days are between 833 and 1250 days. An evaluation report on Shenzhen’s epidemic prevention materials in 2021 noted that the medical supplies and hygiene materials used for manual sampling include sampling booths, swab test tubes, disposable caps, medical protective masks, medical protective clothing, medical isolation shoe covers, medical rubber examination gloves, and disinfectant [24]. The cost of consumables for two different sampling methods is shown in Table 2.

**Table 2:** Consumable items and costs of different sampling methods.

Robot collection			
Consumables item		Average price(¥)	
Equipment Cost and Depreciation		630,000	
Swab test Tube		3 /Piece	
Disinfectant		26.8 /Bottle	
Manual collection			
Consumables item	Average price(¥)	Consumables item	Average price(¥)
Disposable Hat	0.45/ Piece	Isolation shoe Cover	6.7/Pair
Protective Mask	6.05/ Piece	Testing Gloves	0.96/ Pair
Protective Suit	51/ Piece	PE Gloves	0.1/ Pair
Isolation Mask	4.4/ Piece	Disinfectant	26.8 /Bottle
Swab test Tube	3/ Piece	Sampling Booth	35,000/Unit

a.1CNY = 0.16USD

A sampling booth is set up at each sampling point; each medical staff member uses three sets of medical supplies and sanitary materials every day, totaling 289.38 yuan, and receives a subsidy of 300 yuan.

### Statistical test

SPSS software version 22.0 (Armonk, NY: IBM Corp.) was used to analyze the consistency of Ct values of different groups of samples, to analyze whether there was a significant difference between the efficiency of robotic sampling and manual sampling, and to check whether the positive rate of samples in different groups was different.

### Study hypothesis

In conducting the analysis, we made the following assumptions: 1) The aspect of cost mainly included the direct cost of the research, didn’t consider the indirect cost and intangible cost. 2) The number of persons with nucleic acid sample collection was evenly distributed and constant per unit time. 3) Human and robot were calculated according to theoretical working time.

## Results

### Descriptive analysis

A total of 273 volunteers were included for testing and divided into six groups as convenient sample. The basic information of each group is shown in Table 3.

**Table 3:** Basic information of enrolled volunteers.

Group	Sampling method	Number of People		Average Age
		Male	Female	
1	Manual—3 rotations for 5s	38	51	21.28±2.90
2	Manual—3 rotations for 10s	24	6	39.90±11.22
3	Manual—3 rotations for 15s	21	9	40.00±10.85
4	Manual—5 rotations for 10s	24	6	41.17±10.45
5	Manual—5 rotations for 15s	22	8	36.73±10.45
6	Robot—3 rotations for 5s	20	44	29.84±6.65

The results of the questionnaire survey for the sixth group of volunteers (those who underwent robotic sampling) are shown more than 95.31% of respondents knew about nasopharyngeal swab sampling, but nearly half of the respondents (48.44%) reported being fearful of robotic nasopharyngeal swab sampling beforehand, specifically of bleeding or machine failure. After the sampling, 85.94% of the respondents reported that robotic nasopharyngeal swab sampling felt no different or was more comfortable than manual sampling what they’ve experienced before, and 65.62% of the respondents were satisfied with the sampling. Dissatisfied investigators reported that nasal soreness and tearing occurred during robotic nasopharyngeal swab sampling.

### Effectiveness and safety analysis

According to the instructions of the 2019-nCoV nucleic acid detection kit used, test results were valid if the Ct value of the sample was less than 30. The validity results of the Ct value of the nucleic acid detection of 273 volunteers are shown in Table 4.

**Table 4:** Sample efficiency under different sampling methods.

Sampling method	Sample Ct Mean±SD	Is it effective		Total
		Invalid	Valid	
Manual—3 circles for 5s	22.99±1.73	1	88	89
		1.1%	98.9%	100.0%
Manual—3 circles for 10s	23.02±1.81	0	30	30
		0.0%	100.0%	100.0%
Manual—3 circles for 15s	22.85±1.46	0	30	30
		0.0%	100.0%	100.0%
Manual—5 circles for 10s	22.58±1.37	0	30	30
		0.0%	100.0%	100.0%
Manual—5 circles for 15s	23.06±1.23	0	30	30
		0.0%	100.0%	100.0%
Robot—3 circles for 5s	25.28±2.92	2	62	64
		3.1%	96.9%	100.0%
Total		3	270	273
		1.1%	98.9%	100.0%

In all groups, the mean Ct value of all samples was less than 30. The sample efficiency rate of robotic collection method is 96.9%, lower than that of all manual collection methods. The chi-square [25] test value of the sample efficiency of different sampling method groups was 3.751, p = 0.586, meaning that there was no statistically significant difference in the sample efficiency of the six sampling methods, and that the robot automatic nasopharyngeal swab collection showed reliable sample validity.

During the collection process, no serious adverse events occurred with any sampling method. During the robotic sampling process, there may be nasal soreness and tearing, but no adverse reactions such as nasal mucosa bleeding occurred. After robotic sampling, 14.06% of volunteers reported that robotic sampling was less comfortable than manual sampling, but the majority (85.94%) reported that robotic sampling was more comfortable than manual sampling or the two were not much different.

### Cost comparison

Since there were no statistical difference of efficiency between the two methods, so we compared the cost of the two methods in different situation. The cost comparison between manual sampling and robot sampling must consider the ratio of machine workload to manual workload in the application scenario. A Qinggeng robot can complete the whole process of scanning, sampling, and disinfection, which is approximately equal to the on-site work completed by three medical staff members.

#### Cost comparison between single robot sampling and manual sampling

The data show that the number of samples collected per hour for robotic sampling is 40, and the collection can be continued 24 hours a day. For manual sampling, the number of people collected per hour for manual sampling is 100, and the workday is 12 hours. Assume that the number of working days together is t_1_.


$$Robot\;sampling\;cost=R\&D\;expenditure+Equipment\;Cost\;\&\;Depreciation+Sanitary\;materials\;cost=500000+630000+\left(40*3*24*t_{1}+26.8*3*t_{1}\right)=2960.4t_{1}+1130000$$

$$Manual\;sampling\;cost=Sampling\;booth\;Cost+Sanitary\;meterials\;Cost+Subsidies=35000+\left(289.38*3+100*3*12*t_{1}\right)+\left(300*3*t_{1}\right)=5368.14t_{1}+35000$$

**Fig 2:**
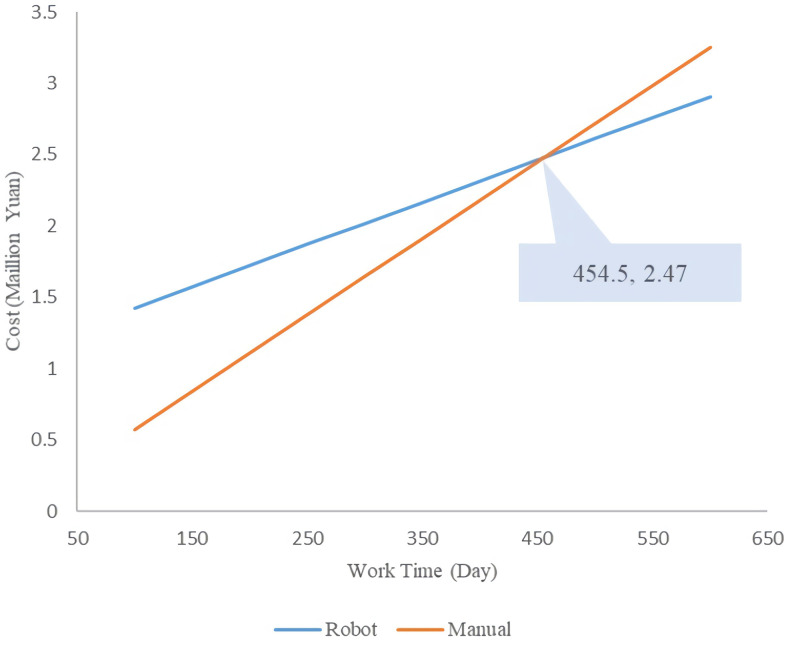
Cost curves over time of single robot sampling and manual sampling.

It is calculated that when t_1_≥455 days, sampling with a single robot will be more economical than manual sampling (Fig 2). Fig 2 shows that if only one nucleic acid sampling point is arranged, when the working time is ≥ 455 days, the cost difference between manual sampling and single machine sampling gradually expands with time.

#### Comparison between multi-robot sampling and manual sampling

Under the condition that the original work efficiency remains unchanged, if more robots are installed for sampling work, within a given time, take t_2_ as an example to compare the costs of the two methods. Now suppose that y robots are put into operation, and 2y medical staff are dedicated to sampling at the same time.


$$Robot\;sampling\;cost=R\&D\;expenditure+Equipment\;Cost\;\&\;Depreciation+Sanitary\;materials\;cost=500000+6300000*y+\left(40*3*24*y+26.8*3*y\right)*t_{2}=2960.4yt_{2}+630000y+500000$$

$$Manual\;sampling\;cost=Sampling\;booth\;Cost+Sanitary\;meterials\;Cost+Subsidies=35000*2y+\left(289.38*2y*3*t_{2}+100*2y*3*12*t_{2}\right)+\left(300*2y*3*t_{2}\right)=70000y+8936.28yt_{2}+1800yt_{2}=70000y+10736.28yt_{2}$$

**Fig 3:**
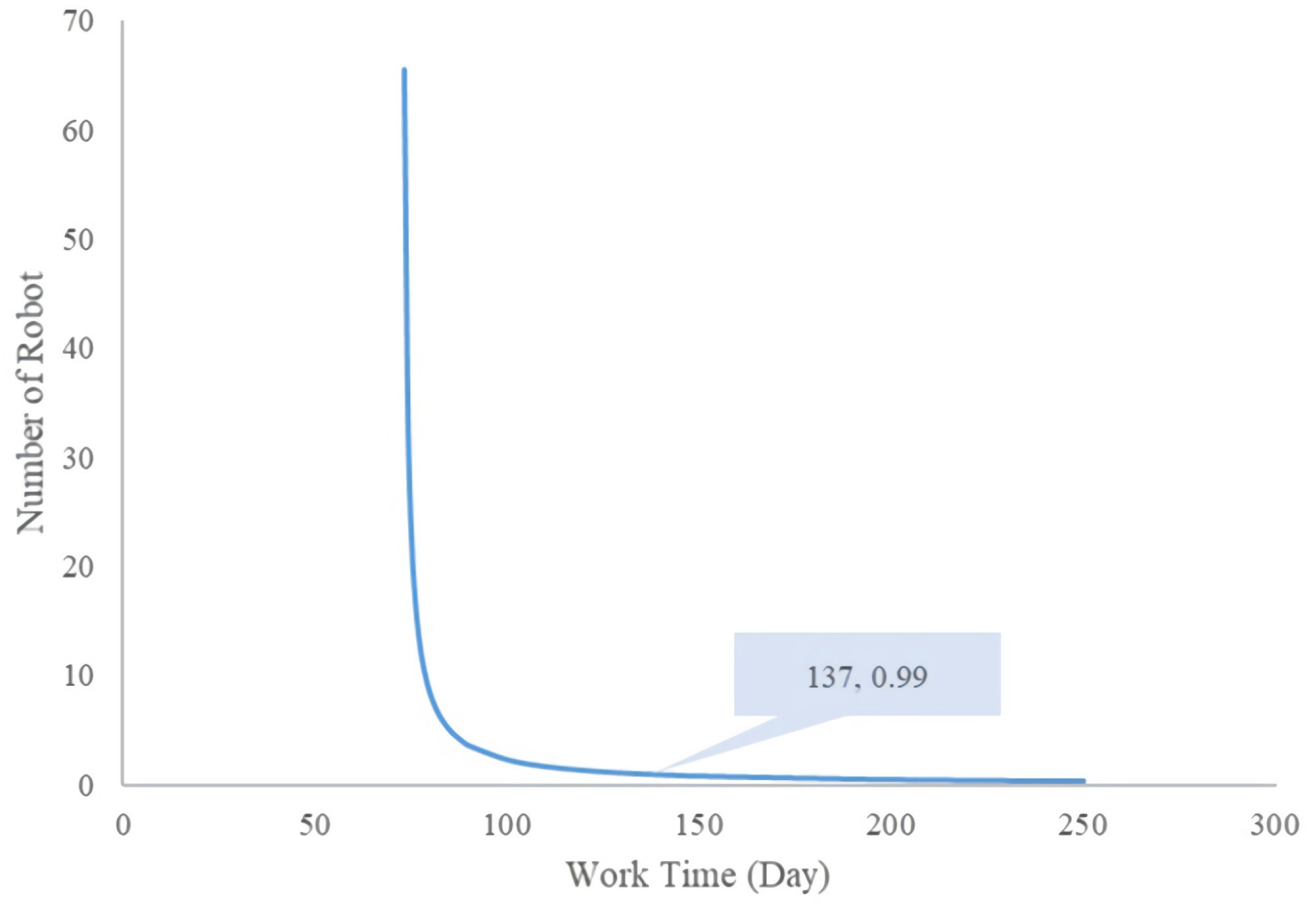
Cost balance curve between multi-robot sampling and manual sampling.

It is calculated that t_2_ should not be less than 73 days. When t_2_ is less than 73 days, the marginal cost of using robot sampling will be higher than that of manual sampling [26]. In hypothetical examples when the robot and labor work together for 73 days, when y≤65, the cost of using robot sampling will be higher than the cost of manual sampling. In this case, if the robot and the human work together for 137 days, when y≤0.99, the cost of using robot sampling will be higher than that of manual sampling; this means that if the working time is 137 days or longer, using the robot to collect samples will be more economical than manual collection (Fig 3).

## Discussion

We found that the sample efficiency of the robotic automatic nasopharyngeal swab sampling method is comparable to that of the manual nasopharyngeal swab sampling method (96.9% VS 98.9%, p = 0.586>0.05), with no statistically significant difference between the two methods. However, objectively speaking, the sample efficiency rate collected by robots was lower than that of other manual sampling groups. From the perspective of the sample collection process, the sample efficiency rates of each group (Table 4) showed that in the process of nasopharyngeal swab sampling, the sampling cotton swab should remain in the nasal cavity as long as possible to fully contact the virus [27], to avoid the occurrence of ineffective sample collection.

There were no serious adverse events in any of the groups in this study. The questionnaire responses for the robotic sampling group showed that 85.94% of the respondents believed that robotic nasopharyngeal swab sampling was no different or more comfortable than manual sampling. After the sampling, 9.38% of the respondents were very satisfied with the sampling, 56.25% were satisfied, and 34.48% had an average degree of satisfaction. Compared with oropharyngeal swabs, the sampling process of nasopharyngeal swabs reduces the occurrence of vomiting and coughing of the sampled [27, 28], but the sampled person may feel nasal soreness or tearing [29], which is a normal physiological response. Therefore, robotic nasopharyngeal swab sampling appears to also be quite safe, comparable to manual sampling.

The cost analysis results of the two sampling methods showed that, under feasible conditions, it was more cost-effective to use multiple machines to replace manual sample collection when working for a long time. Specifically, when using a single robot and a single person sampling manually, if the working time exceeded 454 days, it became more cost-saving to use the machine for sample collection. In the case of multiple robots and manual sampling with twice the number of workers, if the working time exceeded 137 days, using the robot for collection is more cost-saving than manual collection. Hence, we believed that the more manual workers were replaced by robots, the longer the working time, the more cost-effectiveness to use robots collect nucleic acid samples. However, we made some assumptions to keep the working efficiency of the two groups constant. When calculating the cost, only the physical cost under normal circumstances was considered; the operating cost after implementation was not considered. In addition, certain real-world factors, including the exposure and contagion risks of manual detection were not represented in this analysis also.

Some problems with robot-collected samples were noted: First, different light sources directly affected the imaging quality and effect of the image; it was optimal to choose a white background or medical light for both sides and the background when sampling. Second, the fixed position of the camera may cause occlusion and deformation of the image and need to be optimized.

We made additional observations that contribute to optimal results for sample collection: First, the nasopharyngeal swab should enter the nasal cavity perpendicular to the earlobe to a depth of the distance from the tip of the nose to the earlobe [30]. Second, at present, the Qinggeng robot inserts the swab into the nasal cavity against the inner wall. When encountering a patient with a significantly deviated nasal septum, the sampling will be unsuccessful [29–31]. Therefore, it is necessary to subdivide the nasal shape and nasal cavity, and select multiple modes to sample.

## Conclusions

Our results show that it is safe and cost-effective to replace manual nasopharyngeal swab sampling with a fully automatic nasopharyngeal swab robot. This approach is economical and feasible with a significant cost savings when working for a long time and sampling a large number of people. Therefore, policymakers or enterprise could apply it for reference in places with large demand for nucleic acid testing such as customs, entry-exit and hospitals for a long time, so as to save costs and reduce human resources consumption. However, it remains necessary to improve robot sampling procedures by increasing the time of the swab stay in the nasal cavity and making adjustment to the sampling environment to optimize sample acquisition.

## Supporting information

S1 File Informed consent-Chinese version. (DOC)

S2 File Informed consent-English version. (DOC)

S1 Checklist STROBE statement—Checklist of items that should be included in reports of observational studies. (DOCX)
