# Supplementary material for: A study on the performance and cost-effectiveness of robots in replacing manual nucleic acid collection method: Experience from the COVID-19 pandemic
Source: PLoS One. 2022 Nov 3;17(11):e0276782. doi: 10.1371/journal.pone.0276782 (PMC9632764; doi:10.1371/journal.pone.0276782)
Supplement: S1 File — (DOC) [file pone.0276782.s001.doc]

**Subject informed consent**

Subject name：Safety and effectiveness assessment of automatic nasopharyngeal swab sampling robot

Version number and Version date：202104.05，202107.21

Subject informed consent number and date：202105.05，202107.21

**Subject Signature Page**

**Subject Statement:**

□ I have read the above introduction about this study, and the research doctor has explained the research content to me in detail, and I have no more doubts about the study to consult before signing the informed consent form. On this basis, I voluntarily participate in the clinical study presented in this article, and my decision is based on a full understanding of the possible risks and benefits of participating in this study. In addition, the researcher did not use deception, inducement, coercion, etc. against me to force me to agree to participate in the study, and I know that I can unconditionally withdraw from the study at any stage.

□Due to the subject's incapacity and limited capacity, this informed consent shall be signed by his guardian or legal representative.

Subject Signature：

Date:

Telephone：

**Investigator Statement:**

I confirm that the details of this study have been explained to the patient, particularly the possible risks and benefits of participating in this study.

Investigator Signature：

Date

Telephone：

*Note: This page is the signature page of the subjects. The research doctor will explain the research content and related information to the subjects in detail. The informed consent is signed by the subject/guardian/legal representative and the research doctor who explained it. If the subject has any doubts about the research content, the researcher should immediately explain in detail to the subject. After signing, one original copy will be kept by both the investigator and the subject.*
