# Supplementary material for: A study on the performance and cost-effectiveness of robots in replacing manual nucleic acid collection method: Experience from the COVID-19 pandemic
Source: PLoS One. 2022 Nov 3;17(11):e0276782. doi: 10.1371/journal.pone.0276782 (PMC9632764; doi:10.1371/journal.pone.0276782)
Supplement: S2 File — (DOC) [file pone.0276782.s002.doc]

**受试者知情同意书**

项目名称：全自动鼻咽拭子采样机器人安全性和有效性评估

方案版本号及版本日期：202104.05，20210721

知情同意书版本号及版本日期：202105.05，20210721

尊敬的自愿者：

我们邀请您参加深圳市罗湖区人民医院批准开展的全自动鼻咽拭子采样机器人安全性和有效性评估研究。本研究将在深圳市罗湖医院集团开展，预计将有300名受试者自愿参加。本研究已经得到深圳市罗湖区人民医院伦理委员会的审查和批准。

本须知将提供给您一些信息以帮助您决定是否参加此项临床研究，您是否参加本项研究完全是自愿的，且您的决定将不会影响到您在本院的正常诊疗权益和待遇，请放心！若您选择参加本研究，我们研究团队将在研究过程中尽力保证您的安全和权益！

请您仔细阅读本须知，如有任何疑问请向负责为您讲解知情同意书的研究者提出。

1. 研究背景

2019年末爆发的新冠疫情，已造成全球1.8亿人确诊感染，接近405万人因此疫情而丧生。而核酸检测一直是新冠疫情确诊的金标准，良好质量的核酸样本是核酸筛查和检测的重要保障。由于新冠病毒的强传播、快变异和高致病特点，新冠疫情已经成为迄今为止影响最为严重的公共卫生事件，对鼻咽拭子进行规范采集可以提高下游核酸检测的正确性。开发一种全自动鼻咽拭子采样机器人，可大大减少医护人员的劳动强度，减少高危暴露风险；将有限的医疗资源集中到急危重症的救治中，降低死亡率。

项目组开发研制的全自动鼻咽拭子采样机器人，充分利用医院现有大数据平台和实际临床应用场景，开发全自动无人操作鼻咽拭子采集机器人。实现全程无需人工干预，减少医护人员高风险暴露，提高检测效率的整体解决方案。为验证全自动鼻咽拭子采样机器人样本采集的安全性和有效性，特征集不少于300个志愿者在未来六个月（2021年7月-2021年12月）完成样本采集及分析，并基于样本数据分析改进人工智能算法和路径规划，持续迭代，提高采样机器人的安全性和有效性，为医疗器械注册提供准确数据支持。

1. 研究目的

本研究主要目的是针对迄今为止最为广泛的核酸样本进行量化，评估不同采样方式（力度，位置和采集时长)对被采集人获得的样本的数量及质量，建立鼻咽拭子采集规范，为后续核酸检测提供高质量样本，降低假阴性结果。同时，使用建立起的规范鼻咽拭子采样规范评估全自动鼻咽拭子采样机器人的样本质量，通过样本质量和不良反应反馈，改进算法和采样规划，提高采样机器人的智能化程度，可以进一步提高采样成功率和采样质量，完成全自动采样机器人核酸采样的安全性和有效性评估。

1. 试验对象

**纳入标准：**

**试验①和②为**，年满18周岁小于60周岁的健康居民，无严重疾病

能自主表达、理解本研究目的及意义

自愿参与本研究

**试验③为**，年满18周岁小于60周岁的居民，经过人工采样验证为EB病毒/流感病毒/新冠病毒阳性或阴性的人群

能自主表达、理解本研究目的及意义

自愿参与本研究

**所有自愿者同时满足排除标准：**

参加者最近有鼻部外伤或手术病史

或者有鼻中隔明显偏曲

或有慢性鼻道阻塞

或有严重凝血障碍的病史

或有严重疾病：恶性肿瘤、严重心脑血管疾病、需要进行重大器官移植的手术、有可能造成终身残疾的伤病、晚期慢性病、深度昏迷、永久性瘫痪、严重脑损伤、严重帕金森病和严重精神病等。

1. 研究过程

本研究分为两部分，第一部分是规范人工核酸样本采集的规范，确定最佳的采样位置、样本量和操作方式。另一方面，以人工采样的数据为支撑，完成全自动鼻咽拭子采样机器人采样评判，通过算法持续改进和路径优化，验证全自动鼻咽拭子采样机器人的安全性和有效性；分析采样人群成功率（不同性别，年龄和鼻腔结构），制定不同人群的采样参数和采样机器人的适用人群。

研究过程及入组人群如下：

①邀请1-2位耳鼻喉专家和资深护师对2-4名资深护士进行讲解鼻咽部解剖结构，按照新英格兰医学杂志发布的鼻咽拭子流程视频，对入组志愿者进行样本采集。在未来两个月（2021年7月-2021年9月）完成样本采集及分析，根据停留鼻腔中采样旋转圈数（2圈/3圈/5圈），停留时间长短（5秒/10秒/15秒）的不同组合方式，每组不少于20个自愿者，人工采样每人左右两侧，合计180人次。一侧样本用于RT-PCR检测新冠病毒，获得样本保守基因片段Ct值，推算获得细胞数目；一侧样本接种病原菌培养和鉴定，通过分析数据得到不同组合得到最佳的采样方式和检验科能够检测的标本低值。

②通过前期建立的人工采样流程和标准，对入组志愿者进行全自动鼻咽拭子机器人进行采集，评估机器人采样的安全性和有效性。对于机器人采集样本，比较不同旋转圈数（旋转3圈和6圈）和停留时间（停留5s和10s），总计4个组合，受试者每次测试均需完成机器人采集，每间隔一周完成一次测试，合计80人次，样本用于RT-PCR检测新冠病毒，获得样本保守基因片段Ct值，推算获得细胞数目；培养鉴定病原菌种类；通过鼻咽喉镜检查获得不同条件下的鼻咽部损伤情况，获得全自动鼻咽拭子参数本身的样本质量、不良反应和与人工采集样本的差异性，找到机器人鼻咽拭子采样的合适途径。

③选择EB/流感/新冠病毒鉴定阳性或阴性患者入组，每组至少20人，合计60人次，每个受试者测试2次，一次人工采集，一次机器人采集，采用上游试验①和②得到的人工和机器采样合适路径和参数，比较机器人和人工采集对于鼻咽拭子的定位准确率（记录采样成功率）、操作精度（人工和机器人采样感受对比）、力控大小（机器人后台显示）和采样消耗时间（棉签配准之后开始计时到棉签采样完成时长），检测获得样本的EB病毒/流感病毒/新冠病毒的阳性率，比较人工采集和机器采集的吻合度，验证机器人采样的敏感性和特异性。

1. 替代治疗

不参加本研究的自愿者，可采用常规核酸采样。常规核酸采样方法为人工鼻咽拭子、口咽拭子或肛拭子等，在其他认证医疗机构、本院或本医院集团社康中心亦可完成，一般约24小时内取得报告。

或参加别的研究，比如香港中文大学深圳校区的人机互动口咽拭子采样。

1. 可能的风险与不适

在试验中，可能预见的风险如下：采样过程中鼻咽拭子力量过大造成不良反应，包含出血、红肿、喷嚏和继发感染等不适，此外也可能存在机器故障，机械臂运动中对受试者造成击打、擦伤等物理伤害。经过前期评估，全自动采样机器人发生机器臂故障概率极小，属于机械臂基本功能要求，同时在该机器人中使用了精确力控传感，在碰到气球等值的压力即可逼停机械臂运动，同时也设置了应急按钮，可以在发生故障时第一时间切断电源，保证受试者安全；同时，对于采样区域和机器人运动区域进行有效划分，对于采样过程中的不良反应，可以通过高精度力控传感，减少不良反应的发生，以往的经验可知，其机器人在该算法条件下的力度和不适感接近或低于人工采样，人工采样中出现的不良反应处理方式可以直接应用于机器人，在测试中发生具体不良反应，按照不同等级进行专业医护处理（包含出血、红肿等不良反应）。

1. 预期获益

获得的鼻咽拭子样本在满足医学中心检验质控要求后，可以免费开具受试者感染病原菌（新冠病毒，革兰氏阳性菌/阴性菌或EB病毒等）认证报告。

1. 免费治疗

对于发生的不良反应进行分类，比如严重不良反应，由于机器人失灵或机器故障造成物理损伤，一般不良反应比如出血、红肿和呕吐等，进行必要的免费医疗治疗；对于轻微不良反应如恶心等进行相关原理解释说明，并辅助完成心理疏导。

1. 补偿

对研究受试者参与采样的按照国际伦理相关规范和医院集团医学伦理相关规范给予合理补偿（（计划按照参与机器人采样200元/例给予补贴，仅参与人工采样的100元/例给予补贴），该费用在完成测试后一次性支付。

1. 研究前中后的注意事项

在知情同意签订前，研究者有义务详细讲解试验计划，试验风险及收益，保证受试者满足纳入标准且在排除标准以外，在完成知情同意书签订后，获准参加试验。在试验后，受试者需要按照研究者指示和建议，按照操作手册要求接受试验流程，若出现不能理解或不能配合试验，需要终止进一步研究。

1. 保密性

自愿者参与本实验，有权利阅读试验计划，研究方案，相关获益及风险提示；同时也需要保守本研究的相关内容，不得向第三方提供相关文献资料或相关咨询服务。

对于受试者，采集的数据包含被采集人的身份信息仅保存原始记录，用于溯源查询，在数据分析、展示和论文或专利中均脱敏处理。

采集的生物学样本准确编号，对应原始记录中的身份信息，严格按照相应生物安全级别进行保存、运输、检测和分析，对于需要重复测定的样本或者暂存的样本提交医院集团生物样本存储统一管理进出入库，数据中的个人信息需严格保密，且仅用于本研究。

1. 重新获取知情同意

当研究者研究内容变更或安全信息出现更新时，研究者需要在相关法规要求范围内修改知情同意书和研究方案，提交伦理委员会补充审查，并通知受试者重新签署知情同意书，只有签订知情同意术后方可进入下一步试验。

1. 自愿性

在知情同意过程中，明确告知受试者可以选择不参加本项研究，或者在任何时候通知研究者要求退出研究，受试者数据将不纳入研究结果，受试者的任何医疗待遇与权益不会因此而受到影响；同时受试者可在任意时刻不需要任何理由退出试验，不需负担其他责任。

1. 受试者义务

受试者需准确告知研究者自己的疾病史和身体状态，不得故意隐瞒相关信息；对于受试者在参与试验中出现的任意不良反应或者不舒适感受需要及时告知研究者；同时，受试者若同时参与类似的研究，需要明确告知研究者。

1. 联系方式

如果您有与本研究有关的问题，或您在研究过程中发生了任何不适与损伤，或有关于本项研究参加者权益方面的问题您可以通过19925133889与研究者覃金洲联系。

如果您有任何疑问或在研究过程中对研究人员有抱怨，可以联系深圳市罗湖区人民医院科研伦理委员会，联系方式:0755-82205691。

**受试者签字页**

**受试者同意声明：**

□ 我已经阅读了上述有关本研究的介绍，且研究医生已向我详细地讲解了研究内容，在签署知情同意书前我已没有更多有关研究的疑惑需咨询。在此基础上，我自愿参加本文所介绍的临床研究，并且我的决定是基于对参加本研究可能产生的风险和受益充分了解。此外，研究者没有对我使用欺骗、利诱、胁迫等手段强行让我同意参加研究，并且我知道我可以在任何阶段无条件退出研究。

□该名受试者因无行为能力、限制行为能力，本知情同意由其监护人或者法定代理人代为签署。

受试者签名：

日 期： 年 月 日 时 分

受试者联系方式：

**研究者声明：**

我确认已向患者解释了本研究的详细情况，特别是参加本研究可能产生的风险和收益。

研究者签名：

日 期： 年 月 日 时 分

研究者联系方式：

*注意：本页为受试者签字页，由研究医生向受试者详细讲解研究内容及相关信息，知情同意由受试者本人/监护人/法定代理人及为其讲解的研究医生签署。若受试者对研究内容有疑问，研究者应立即当面向受试者详细解释。签署完毕后，由研究者和受试者双方各保留一份原件。*
