# Supplementary material for: A study on the performance and cost-effectiveness of robots in replacing manual nucleic acid collection method: Experience from the COVID-19 pandemic
Source: PLoS One. 2022 Nov 3;17(11):e0276782. doi: 10.1371/journal.pone.0276782 (PMC9632764; doi:10.1371/journal.pone.0276782)
Supplement: S1 Checklist — (DOCX) [file pone.0276782.s003.docx]

STROBE Statement—checklist of items that should be included in reports of observational studies

|  | Item No. | Recommendation | Page  No. | Relevant text from manuscript |
| --- | --- | --- | --- | --- |
| **Title and abstract** | 1 | (*a*) Indicate the study’s design with a commonly used term in the title or the abstract | 2 | For this observational research… |
|  |  | (*b*) Provide in the abstract an informative and balanced summary of what was done and what was found | 2 | See in results and conclusions. |
| Introduction | | | |  |
| Background/rationale | 2 | Explain the scientific background and rationale for the investigation being reported | 3 | Since the outbreak of the novel coronavirus-2019 (COVID-19) pandemic，RT-PCR detection has been the primary detection method for diagnosing the disease because of its early diagnosis, good sensitivity, and high specificity… |
| Objectives | 3 | State specific objectives, including any prespecified hypotheses | 4-5 | Hangzhou Huxi Yunbaisheng Technology Co., Ltd. has developed the world's first fully automatic nasopharyngeal swab sampling robot, named “Qinggeng”. We sought to explore the differences between manual swab collection and robotic swab collection in the COVID-19 era, evaluate the safety, effectiveness and economy of robotic sample collection, and provide a research basis for the use of robots in such situations… |
| Methods | | | |  |
| Study design | 4 | Present key elements of study design early in the paper | 5-10 | See subtitles in methods. |
| Setting | 5 | Describe the setting, locations, and relevant dates, including periods of recruitment, exposure, follow-up, and data collection | 5 | Luohu Hospital sent research information online and healthy volunteers who find the information were recruited in December 2021… |
| Participants | 6 | (*a*) *Cohort study*—Give the eligibility criteria, and the sources and methods of selection of participants. Describe methods of follow-up  *Case-control study*—Give the eligibility criteria, and the sources and methods of case ascertainment and control selection. Give the rationale for the choice of cases and controls  *Cross-sectional study*—Give the eligibility criteria, and the sources and methods of selection of participants | 6 | See Inclusion and Exclusion Criteria. |
|  |  | (*b*) *Cohort study*—For matched studies, give matching criteria and number of exposed and unexposed  *Case-control study*—For matched studies, give matching criteria and the number of controls per case |  |  |
| Variables | 7 | Clearly define all outcomes, exposures, predictors, potential confounders, and effect modifiers. Give diagnostic criteria, if applicable | 5-6 | After sample collection, the Ct value of each sample was detected and compared with the internal standard Ct value. Sample Ct values of less than the internal standard Ct value were judged to be valid; higher values were considered invalid…/ SPSS software version 22.0 (Armonk, NY: IBM Corp.) was used to analyze the consistency of Ct values of different groups of samples, to analyze whether there was a significant difference between the efficiency of robotic sampling and manual sampling, and to evaluate the effectiveness of robotic sampling…/ After sample collection, volunteers in the robotic collection group were asked to complete an additional questionnaire about robotic sampling to record their impressions of the process for analysis of the difference between robotic sampling and manual sampling…/ The cost components of the two different collection methods were also collected to analyze and predict the economic factors involved… |
| Data sources/ measurement | 8* | For each variable of interest, give sources of data and details of methods of assessment (measurement). Describe comparability of assessment methods if there is more than one group | *7-10* | *See collection process and cost part.* |
| Bias | 9 | Describe any efforts to address potential sources of bias | N/A | N/A |
| Study size | 10 | Explain how the study size was arrived at | N/A | N/A |

Continued on next page

| Quantitative variables | | 11 | | Explain how quantitative variables were handled in the analyses. If applicable, describe which groupings were chosen and why | 5,10 | | Volunteers were divided into six groups according to the type of sampling and the duration time for manual sampling…/ A total of 273 volunteers were included for testing and divided into six groups as convenient sample… |
| --- | --- | --- | --- | --- | --- | --- | --- |
| Statistical methods | | 12 | | (*a*) Describe all statistical methods, including those used to control for confounding | 5,12 | | SPSS software version 22.0 (Armonk, NY: IBM Corp.) was used to analyze the consistency of Ct values of different groups of samples…/ The chi-square [25] test value of the sample efficiency of different sampling method groups was 3.751… |
|  |  |  |  | (*b*) Describe any methods used to examine subgroups and interactions | N/A | | N/A |
|  |  |  |  | (*c*) Explain how missing data were addressed | N/A | | N/A |
|  |  |  |  | (*d*) *Cohort study*—If applicable, explain how loss to follow-up was addressed  *Case-control study*—If applicable, explain how matching of cases and controls was addressed  *Cross-sectional study*—If applicable, describe analytical methods taking account of sampling strategy | N/A | | N/A |
|  |  |  |  | (*e*) Describe any sensitivity analyses | N/A | | N/A |
| Results | | | | | | | |
| Participants | | 13* | | (a) Report numbers of individuals at each stage of study—eg numbers potentially eligible, examined for eligibility, confirmed eligible, included in the study, completing follow-up, and analysed | 10 | | A total of 273 volunteers were included for testing and divided into six groups as convenient sample. |
|  |  |  |  | (b) Give reasons for non-participation at each stage |  | |  |
|  |  |  |  | (c) Consider use of a flow diagram |  | |  |
| Descriptive data | | 14* | | (a) Give characteristics of study participants (eg demographic, clinical, social) and information on exposures and potential confounders | 10 | | See in Table 2. |
|  |  |  |  | (b) Indicate number of participants with missing data for each variable of interest | N/A | | N/A |
|  |  |  |  | (c) *Cohort study*—Summarise follow-up time (eg, average and total amount) | N/A | | N/A |
| Outcome data | | 15* | | *Cohort study*—Report numbers of outcome events or summary measures over time | N/A | | N/A |
|  |  |  |  | *Case-control study—*Report numbers in each exposure category, or summary measures of exposure | N/A | | N/A |
|  |  |  |  | *Cross-sectional study—*Report numbers of outcome events or summary measures | *11-14* | | *See Table 3. Figure1,Figure2.* |
| Main results | | 16 | | (*a*) Give unadjusted estimates and, if applicable, confounder-adjusted estimates and their precision (eg, 95% confidence interval). Make clear which confounders were adjusted for and why they were included | 11-14 | | The sampling efficiency of the robot group was 96.9%, and there was no statistically significant difference between the other five manually sampled groups (p=0.586). There were no serious adverse events in any of the six groups, but nasal soreness and tearing did occur. Of the volunteers who underwent robotic sampling, 85.94% reported that the experience was either no different or more comfortable than the manual sampling. In economic terms, a single robot used to replace medical staff for sample collection becomes economically advantageous when the working time is ≥ 455 days. If multiple robots are used to replace twice the number of manual collections, it becomes more economical at 137 days and remains so as long as the robot is used. |
|  |  |  |  | (*b*) Report category boundaries when continuous variables were categorized | N/A | | N/A |
|  |  |  |  | (*c*) If relevant, consider translating estimates of relative risk into absolute risk for a meaningful time period | N/A | | N/A |
| Other analyses | 17 | | Report other analyses done—eg analyses of subgroups and interactions, and sensitivity analyses | | N/A | N/A | |
| Discussion | | | | | | | |
| Key results | 18 | | Summarise key results with reference to study objectives | | 17 | It is safe and effective to replace manual nasopharyngeal swab sampling with a fully automatic nasopharyngeal swab robot, which can greatly reduce the labor intensity of medical staff, reduce the risk of exposure and transmission, and concentrate limited medical resources for the treatment of acute and critical illness. This approach is economical and feasible with a significant cost savings when working for a long time and sampling a large number of people. | |
| Limitations | 19 | | Discuss limitations of the study, taking into account sources of potential bias or imprecision. Discuss both direction and magnitude of any potential bias | | 16 | However, when analyzing economics, we assumed that the number of samples per hour for robot sampling and manual sampling remains constant. When calculating the cost, only the physical cost under normal circumstances was considered; the operating cost after implementation was not considered…/ Some problems with robot-collected samples were noted… | |
| Interpretation | 20 | | Give a cautious overall interpretation of results considering objectives, limitations, multiplicity of analyses, results from similar studies, and other relevant evidence | | 16-17 | We made additional observations that contribute to optimal results for sample collection… | |
| Generalisability | 21 | | Discuss the generalisability (external validity) of the study results | | N/A | N/A | |
| Other information | | |  | | | | |
| Funding | 22 | | Give the source of funding and the role of the funders for the present study and, if applicable, for the original study on which the present article is based | | N/A | N/A | |

*Give information separately for cases and controls in case-control studies and, if applicable, for exposed and unexposed groups in cohort and cross-sectional studies.

**Note:** An Explanation and Elaboration article discusses each checklist item and gives methodological background and published examples of transparent reporting. The STROBE checklist is best used in conjunction with this article (freely available on the Web sites of PLoS Medicine at http://www.plosmedicine.org/, Annals of Internal Medicine at http://www.annals.org/, and Epidemiology at http://www.epidem.com/). Information on the STROBE Initiative is available at www.strobe-statement.org.
